# Evaluation of platelet activity by multiple electrode impedance aggregometry in acute coronary syndromes: pilot study in a Brazilian tertiary-care public hospital

**DOI:** 10.1590/1414-431X20188001

**Published:** 2019-01-10

**Authors:** A. De Lorenzo, M. Dutra, M.A. de Mattos, H.C.V. Rey, E. Tibirica

**Affiliations:** Instituto Nacional de Cardiologia, Ministério da Saúde, Rio de Janeiro, RJ, Brasil

**Keywords:** Dual antiplatelet therapy, P2Y_12_ antagonists

## Abstract

There is no definite recommendation for testing platelet aggregation (PA) in acute coronary syndromes (ACS) due to inconclusive evidence on the usefulness of platelet function tests to guide therapy and improve clinical outcomes. The evaluation of PA with multiple electrode impedance platelet aggregometry (MEA) may be useful to manage antiplatelet therapy and possibly influence patient outcome. The primary aim of this study was to measure PA with MEA in Brazilian patients with ACS and evaluate the association between PA and adverse clinical outcomes. Forty-seven consecutive patients admitted with ACS to a Brazilian tertiary-care public hospital were studied and PA was evaluated using MEA. Patients were followed for six months for the occurrence of all-cause death, acute myocardial infarction, or stroke. Suboptimal inhibition of PA was found in 7 patients (14.9%); 5 (10.6%) in response to ASA (acetylsalicylic acid), 2 (5.0%) to clopidogrel, and none to ticagrelor. Inadequate PA inhibition in response to ASA was significantly associated with the composite end point, but there was no significant association for insufficient PA inhibition in response to clopidogrel. This study suggested that the evaluation of PA in ACS using MEA may identify non-responders to ASA. Larger studies are necessary to define, in a public health scenario, the value of MEA in the management of ACS.

## Introduction

Dual antiplatelet therapy with acetylsalicylic acid (ASA) plus a P2Y_12_ receptor antagonist (clopidogrel, prasugrel, or ticagrelor) is the cornerstone of the treatment of acute coronary syndromes (ACS) (1). However, about one-third of patients do not achieve adequate inhibition of platelet aggregation (PA) (2–4), resulting in an increased risk of thrombotic events. Nonetheless, there is no definite recommendation in the literature for testing PA in ACS (5,6) due to prior inconclusive evidence on the usefulness of platelet function tests (PFT) to guide therapy and improve outcomes. Nevertheless, a recent Hungarian study has shown that, in patients with acute myocardial infarction, treatment guided by MEA-switching P2Y_12_ antagonists was associated with lower mortality (7). Additionally, while the assessment of PA has been traditionally considered a limitation in itself, because classic platelet aggregometry is difficult to perform, time-consuming, and prone to a variety of problems due to pre-analytical variables (8), whole-blood multiple electrode impedance platelet aggregometry (MEA) is less complex and more reproducible (9). In Brazil, the most frequently used P2Y_12_ antagonist is clopidogrel, for financial reasons. In a developing country with universal public access to healthcare and significant budget restrictions, the use of MEA for guided antiplatelet therapy, with more costly P2Y_12_ antagonists targeted to patients with suboptimal PA inhibition with clopidogrel, might therefore optimize patient management and costs.

Considering that platelet aggregability has already been demonstrated to be associated with hard endpoints in ACS (10,11), and even though the use of PFT to guide therapy in patients with ACS is still a controversial area under discussion, there are no data regarding this association in our country.

Thus, this pilot study sought to investigate PA levels with MEA in patients with ACS admitted to a tertiary-care public hospital in Rio de Janeiro, Brazil.

## Material and Methods

This was a prospective observational study that included consecutive patients during a nine-month period (from October 2015 to July 2016) admitted with ACS at the National Institute of Cardiology in Rio de Janeiro, Brazil. The study complied with the 1975 Declaration of Helsinki and was approved by the Institutional Review Board (#CAAE 36531914.7.0000.5272). All patients signed an informed consent form.

The criteria used to define cardiovascular risk factors were obtained from the following guidelines: *i*) hypertension, from the 7th Brazilian Guidelines of Arterial Hypertension (12); *ii*) diabetes mellitus, from the Classification and Diagnosis of Diabetes (13); *iii*) dyslipidemia, from the Third Report of the National Cholesterol Education Program (NCEP) Expert Panel on Detection, Evaluation, and Treatment of High Blood Cholesterol in Adults (Adult Treatment Panel III) Final Report (14); *iv*) obesity, from the World Health Organization, body mass index (BMI) classification (http://apps.who.int/bmi); *v*) smoking, from the Smoking Cessation and the Risk of Cardiovascular Disease Outcomes Predicted from Established Risk Scores: Results of the Cardiovascular Risk Assessment among Smokers in Primary Care in Europe (CV-ASPIRE) study (15).

Exclusion criteria were active bleeding, shock, end-stage malignant diseases, kidney failure with dialysis therapy, or any contraindication for dual antiplatelet therapy. GRACE, CRUSADE, and TIMI scores were evaluated, and patients were stratified into risk categories according to previously described criteria. Initially, 52 patients were selected for the study; nevertheless, five patients who were submitted to coronary artery bypass grafting and were treated only with ASA were excluded from the study. Thus, all remaining study patients received dual antiplatelet therapy according to current guidelines (16), with ASA plus clopidogrel, with loading doses of 200 and 300 mg, respectively, followed by 100 mg and 75 mg daily, respectively. Ticagrelor was reserved for high-risk patients according to the GRACE score or for suspected clopidogrel resistance (occurrence of thrombotic events during therapy with ASA plus clopidogrel). In that situation, patients received initially 100 mg of ASA + 180 mg of ticagrelor, followed by 100 mg of ASA daily + 90 mg of ticagrelor twice a day.

Whole-blood PA was evaluated, using MEA (Multiplate®, Roche Diagnostics GmbH, Switzerland), in the first 72 h after the diagnosis of ACS. Even if platelet aggregability may vary with time, after a loading dose of clopidogrel, platelet aggregation decreases and remains stable over at least 48 h (17). The evaluation of PA inhibition by P2Y_12_ antagonists was performed by the quantification of the area under the aggregation curve (AUC) induced by adenosine diphosphate, and adequate PA inhibition was defined as values ≤46 U. The evaluation of PA inhibition by ASA was performed by quantification of the AUC induced by arachidonic acid, and adequate PA inhibition was defined as values ≤40 U, according to the manufacturer and to previous clinical studies (18–20).

Patients were evaluated three and six months after discharge. If patients missed any scheduled consultation, outcome data were collected through telephone contact. A composite endpoint consisting of all-cause death, acute myocardial infarction, or stroke during the 6-month follow-up was evaluated.

Categorical variables are reported as frequencies and percentages and compared using chi-squared or Fisher's exact test. Continuous data are reported as means±SD (parametric data) or median and interquartile range (nonparametric data), according to the Shapiro-Wilk test. P<0.05 was considered significant.

## Results

Forty-seven patients were studied (Table 1). In general, patients were of intermediate risk according to GRACE, TIMI, and CRUSADE scores. All patients received ASA, while 40 (85.1%) received clopidogrel and 7 (14.9%) received ticagrelor. Seven patients (14.9%) had inadequate PA inhibition: five patients (10.6%) in response to ASA and two to clopidogrel (5.0%). None of the patients had suboptimal PA inhibition in response to ticagrelor.

**Table 1:** Clinical characteristics of patients (n=47).

	Number	Percentage
Male sex	29	61.7
Risk factors		
Hypertension	37	78.7
Diabetes Mellitus	19	40.4
Dyslipidemia	20	42.6
Current smoking	15	31.9
Prior smoking	20	42.6
Family history of CAD	7	14.9
Obesity	11	23.4
Prior MI	16	34.0
Prior CABG	6	12.8
Prior PCI	13	27.7
Left ventricular function		
Moderate or severe dysfunction*	11	23.4
Diagnosis at hospital admission		
Unstable angina	21	44.7
STEMI	13	27.7
NSTEMI	13	27.7
Medication use		
ASA	47	100.0
Clopidogrel	40	85.1
Ticagrelor	7	14.9
Enoxaparin	43	91.5
Myocardial reperfusion		
PCI	25	53.2
Thrombolytic therapy	10	21.3
CABG	4	8.5
Risk scores		
TIMI	4.0 (2.7–5.0)	
GRACE	115.0 (90.2–144.5)	
CRUSADE	34.5 (22.7–48.5)	

Parametric data are reported as means±SD and non-parametric data are reported as the median (interquartile range). ASA: acetylsalicylic acid; CABG: coronary artery bypass grafting; CAD: coronary artery disease; GRACE: Global Registry of Acute Coronary Events; CRUSADE: Can Rapid Risk Stratification of Unstable Angina Patients Suppress Adverse Outcomes with Early Implementation; PCI: percutaneous coronary intervention; MI: myocardial Infarction; STEMI: ST-elevation myocardial infarction; NSTEMI: Non-ST-elevation myocardial infarction; TIMI: thrombolysis in myocardial infarction. *<45% left ventricular ejection fraction (transthoracic echocardiogram).

During follow-up, there were seven events (14.9%) (Table 2). This rather high number of events is probably related to the fact that patients of our sample were of high cardiovascular risk, as shown by the risk scores. Moreover, most patients were transferred from primary health care units to our tertiary care unit, which is dedicated to cardiovascular care in high-complexity patients and cardiovascular procedures. Inadequate PA in response to ASA was significantly associated with composite events (60 *vs* 40% in patients with therapeutic levels of PA inhibition, P=0.006). On the other hand, there was no significant association with suboptimal PA in response to clopidogrel (50% of events with or without therapeutic PA inhibition, P=0.2). Of note, no patient had any significant bleeding, either during hospitalization or after hospital discharge.

**Table 2:** Follow up of adverse events (six months).

Clinical event	Number	Percentage
MI	2	4.3
Stroke	1	2.1
All-cause mortality	4	8.5

MI: myocardial Infarction.

## Discussion

Despite being theoretically useful to identify ACS patients with suboptimal platelet inhibition, in whom therapeutic adjustments are warranted to minimize ischemic complications (19), PFT are currently not recommended for routine use (6). For instance, large studies such as ANTARCTIC (21), performed in elderly patients presenting with ACS who were at high risk of ischemic and bleeding complications, do not support the use of PFT to guide the choice of antiplatelet drugs and their doses. This still controversial issue is even more relevant in countries with limited access to newer antiplatelet agents and to laboratory technology for the measurement of PA. This pilot study was therefore designed to evaluate, in a patient sample with ACS from a specialized, public Brazilian hospital, the level of PA using MEA and its association with 6-month outcomes.

The frequency of suboptimal PA inhibition observed during this study (14.9%) was similar to that previously reported (22–24). Interestingly, none of the patients receiving ticagrelor had inadequate PA inhibition. In line with other studies (19,21), we did not find any association between suboptimal PA inhibition with clopidogrel and adverse events. However, there was a statistically significant association between inadequate PA inhibition in response to ASA and the composite endpoint. This is contrary to the results of Ibrahim et al. (23), but patient populations were different, as they enrolled only patients with stable coronary artery disease.

This study was limited by sample size and the small number of events, which preclude the analysis of confounding variables associated with endpoints. Nonetheless, regarding PA levels in response to clopidogrel and outcomes, we found negative results that are mostly in line with the literature. On the other hand, the significant association between inadequate PA inhibition in response to ASA and the composite endpoint might indicate that PFT can identify a subgroup of patients who benefit from dose adjustments of ASA (25). Larger studies in the scenario of public health are needed to define if PFT is cost-effective for widespread application.

In conclusion, this study suggested that the evaluation of PA in ACS using MEA might identify non-responders to ASA. Larger studies are necessary to define, in a public health scenario, the value of MEA in the management of ACS. We anticipate that the results will lead to the continuation of the study towards larger patient numbers, and might also serve as further evidence towards the incorporation of this method into clinical practice, especially if confirmed in a larger trial.
